# The Effect of Post-Mastectomy Radiotherapy in Patients With Metaplastic Breast Cancer: A Propensity Score-Matched Analysis of the SEER Database

**DOI:** 10.3389/fonc.2021.593121

**Published:** 2022-01-12

**Authors:** Jin Hu, Jie Tan, Fang Dong, Ximeng Zhang, Jie Ming, Tao Huang

**Affiliations:** Department of Breast and Thyroid Surgery, Union Hospital, Tongji Medical College, Huazhong University of Science and Technology, Wuhan, China

**Keywords:** radiotherapy, survival, SEER, metaplastic breast cancer, propensity score-matching

## Abstract

**Background:**

Metaplastic breast cancer (MBC) is a rare tumor with aggressive biological behavior. This study aimed to evaluate the efficacy of post-mastectomy radiotherapy (PMRT) on patients with low-risk (T1N0M0), intermediate-risk (T1-2N1M0 and T3N0M0), and high-risk (T1-4N2-3M0 and T4N0-1M0) MBC *via* propensity-score matching (PSM).

**Methods:**

We analyzed information from the Surveillance, Epidemiology, and End Results (SEER) public-use database from 1975 to 2016 for MBC incidence trends and compared overall survival (OS) and breast cancer-specific survival (BCSS) between groups of MBC women diagnosed from 2001 to 2016 using Kaplan–Meier analysis and the multivariate Cox proportional model. PSM was used to make 1:1 case–control matching.

**Results:**

Joinpoint analyses identified 1984 and 2003 as the inflection points among 4,672 patients. 1,588 (42.4%) of the 3,748 patients diagnosed with MBC between 2001 and 2016 received PMRT. According to multivariate analyses, PMRT provided better OS (p < 0.001) and BCSS (p < 0.001) before PSM, and better prognosis after PSM (n = 2528) for patients receiving PMRT (n = 1264) compared to those without PMRT (OS, p < 0.001 and BCSS, p < 0.001). When stratifying the case–control matching patients into low-risk, intermediate-risk, and high-risk groups, PMRT could improve BCSS compared with that in non-PMRT patients in the high-risk groups; it also improved OS in both the intermediate- and high-risk groups.

**Conclusions:**

Per findings of the PSM analysis, PMRT could provide better BCSS in high-risk groups, and better OS in intermediate- and high-risk groups.

## Background

The World Health Organization recognized metaplastic breast cancer (MBC) as a unique histologic subtype (1). It was in 1987 that the incidence of MBC (0.5%–2%) was mentioned firstly (2). Nowadays, it was still acceptable for researchers (3, 4). Characteristics of MBC are that glandular epithelial cells transformed into other cell types, either a mesenchymal cell type (e.g., chondroid, osseous, spindle cells, and myoid) or a non-glandular epithelial cell type (e.g., squamous cells) (5). Wargotz classified MBC into five subtypes: squamous cell carcinoma, spindle cell carcinoma, carcinosarcoma, matrix-producing carcinoma, and metaplastic carcinoma with osteoclastic giant cells (6–10).

While the National Comprehensive Cancer Network (NCCN) guidelines have recently been revised, MBC management remained largely paralleled that of infiltrating ductal carcinoma (IDC) (11), which is not helpful, given that the prognosis of MBC was so worse than that of IDC. The 5-year survival rate prognosis for MBC ranged from 49% to 83% (3, 12–14).

Using post-mastectomy radiotherapy (PMRT) to manage MBC is a possibility that has yet to gain a unanimous consensus among experts. While some experts have found that patients undergoing PMRT had a better prognosis than their non-PMRT-receiving counterparts (14–21), others have established no connection between PMRT and outcomes (20, 22–24). However, these contrasting findings could yet be explained; they may have stemmed from different treatment patterns and sample sizes of the research populations used by the various researchers. Research methods applied may also have factored in the differences, as could the study variables included, which in this case were incomplete.

To identify a comprehensive treatment approach for MBC, PMRT must be evaluated in-depth and urgently. Therefore, we extracted information from an extensive database, the Surveillance, Epidemiology, and End Results (SEER) registry, and use propensity score matching (PSM) and conventional methods to explore the effect of PMRT in the MBC patients.

## Materials and Methods

### Database and Population

We extracted the data of patients diagnosed with MBC confirmed by pathology between 1975 and 2016 from the SEER database, which contains demographic, clinical, and pathological information and covers approximately 28% of the United States catchment area. Metaplastic histology was identified with ICD-0–3 codes: 8560, 8562, 8570–8572, 8575, and 8980–8982. Patient information was classified into a low-risk group, which included patients at stages T1-2N0M0, an intermediate-risk group which included patients at stages T1-2N1M0 and T3N0M0 (25), and the high-risk group which included patients at stages T1-4N2-3M0 and T4N0-1M0 (26). This study was exempt from the approval processes of the Institutional Review Boards because the SEER database patient information is de-identified.

### Study Variables

The following demographics and clinical and pathological features were extracted: age, insurance status, race, histopathological subtypes, tumor grade, estrogen receptor (ER) status, progesterone receptor (PR) status, chemotherapy record, tumor size, and post-mastectomy radiotherapy record. According to the pathological stage of lymph nodes, we divided them into four groups using the number of lymph node metastasis. We did not include the state of HER-2, because SEER only recorded these data after 2010. The primary endpoints were overall survival (OS) and breast cancer-specific survival (BCSS).

### Joinpoint Analysis and Propensity Score Matching

We used joinpoint analysis to identify the time points of incidence rate changes (27), and the annual percentage change (APC) to characterize resulting trends. We created a matching dataset, using age (over and equal or under 50 years old), tumor grade, tumor size, lymph node status, ER status, PR status, and chemotherapy record (yes versus no) as covariates and employed a 1:1 closest propensity score using PSM to match pairs between the PMRT and non-PMRT groups (match tolerances = 0.0005)

### Statistical Analysis

We used the SPSS statistical software (version 22.0; IBM Corporation, Armonk, NY, USA) for statistical analyses, evaluating the differences between groups using the chi-square test, employing the Kaplan–Meier analysis, and log-rank test for BCSS curves, and assessing risk factors for OS and BCSS using the Cox proportional hazards model. A value of p < 0.05 was defined statistically significant.

## Results

### Incidence of MBC Combined for Men and Women

**Figure 1:**
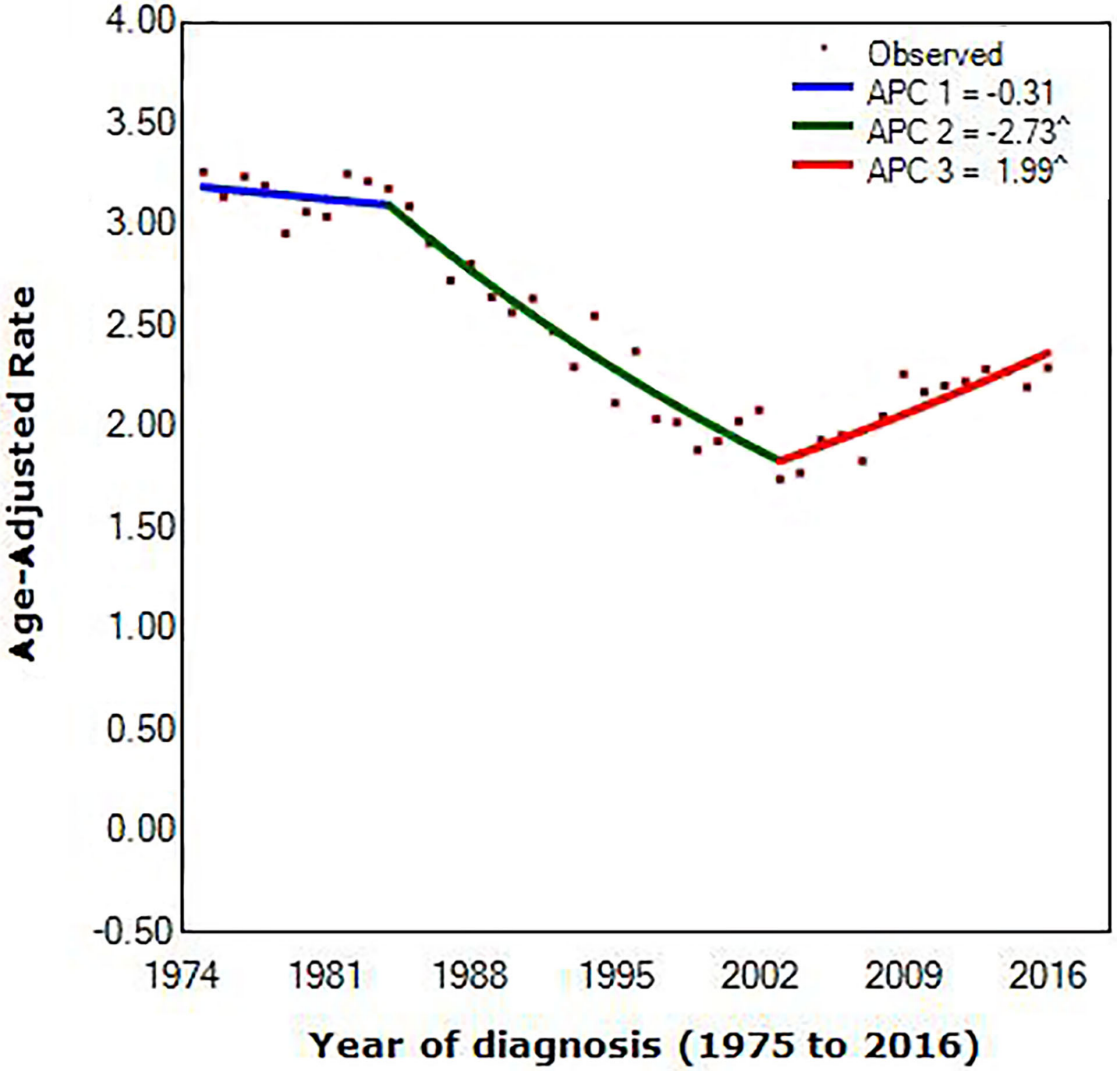
Joinpoint regression of diagnosis of metaplastic breast cancer, by year. (^) p < 0.05; APC, annual percentage change.

We extracted data diagnosed with MBC from the SEER database, counting for 4,672. **Figure 1** showed trends in age-adjusted incidence for MBC combined for men and women from 1975 to 2016. Scattered points represent observed rates. According to joinpoint regression, lines were fitted rates.

Joinpoint occurring in 1984 [95% confidence interval (CI), 1982 to 1986] and 2003 (95% CI, 2002 to 2004) provided the optimal fit of the data, reflecting the years in which average annual MBC incidence rates shifted most markedly during the approximately 40 years scrutinized (1975 to 1984 vs. 1984 to 2003 vs. 2003 to 2016). Incidence of MBC decreased before 2003, with the decrease accelerating from 0.31% (95% CI, –0.7 to 0.1) per year during the 1975 to 1984 period to 2.73% (95% CI, –2.9 to –2.6) per year during the 1984 to 2003 interval. In contrast, incidence rates increased between 2003 and 2016, with a speed of increase of 1.99% (95% CI, 1.7 to 2.2) per year.

### Patient Characteristics

The exclusion criteria after joinpoint analysis are given in **Table 1**. 3,748 patients were derived into two groups (PMRT group and non-PMRT group) based on whether they receipted PMRT or not. **Table 2** shows the characteristics of the patients in our study.

**Table 1:** Stepwise inclusion and exclusion counts.

Removal criterion	Removed	Remaining
1975 to 2016 MBC patients	0 (0.0%)	4,672
Exclude men	9 (0.2%)	4,663
Exclude patients younger than >18 years and <90 years	132 (2.8%)	4,531
Exclude patients who did not receive a mastectomy or lumpectomy	515 (11.3%)	4,016
Exclude those with unknown tumor size or if size = 0	8 (0.2%)	4,008
Exclude patients with borderline ER	11 (0.3%)	3,997
Exclude patients with borderline PR	7 (0.2%)	3,990
Excluded patients without receipt of combination of beam with implants or isotopes; radiation, NOS method or source not specified; radioactive implants (includes brachytherapy)	62 (1.6%)	3,928
Exclude patients who did not have a histologically confirmed diagnosis	12 (0.3%)	3,916
Exclude patients who received neoadjuvant radiation or radiation status unknown	24 (0.6%)	3,892
Year of diagnose after 2000	144 (3.7%)	3,748
Final data set	0 (0.0%)	3,748

MBC, metaplastic breast cancer; ER, estrogen receptor; PR, progesterone receptor; TNBC, triple negative breast cancer.

**Table 2:** Patient demographic and clinical characteristics.

Variables	Before PSM			After PSM		
	Non-PMRT	PMRT	p	Non-PMRT	PMRT	p
	N = 2160	N = 1588		N = 1264	N = 1264	
Age, years			<0.001			0.485
20–30	20 (0.9)	23 (1.4)		16 (1.3)	13 (1.0)	
31–40	103 (4.8)	106 (6.7)		73 (5.8)	79 (6.3)	
41–50	292 (13.5)	261 (16.4)		196 (15.5)	181 (14.3)	
51–60	459 (21.3)	402 (25.3)		289 (22.9)	322 (25.5)	
61–70	537 (24.9)	392 (24.7)		326 (25.8)	319 (25.2)	
71–80	465 (21.5)	271 (17.1)		255 (20.2)	227 (18.0)	
81–90	284 (13.1)	133 (8.4)		109 (8.6)	123 (9.7)	
Follow-up, months [median]	39 (1–191)	49 (1–190)	<0.001	41 (1–191)	51 (1–190)	<0.001
Race/ethnicity			0.037			0.781
Blank	331 (15.3)	288 (18.1)		211 (16.7)	195 (15.4)	
White	1659 (76.8)	1191 (75.0)		979 (77.5)	994 (78.6)	
Asian or Pacific Islander	152 (7.0)	90 (5.7)		66 (5.2)	65 (5.1)	
American Indian/Alaska Native	13 (0.6)	10 (0.6)		8 (0.6)	8 (0.6)	
Unknown	5 (0.2)	9 (0.5)		–	–	
Insurance status			0.218			0.262
Medicaid	218 (10.1)	135 (8.5)		129 (10.2)	106 (8.4)	
Insured	1246 (57.7)	946 (59.6)		753 (59.6)	758 (60.0)	
Uninsured/unknown	696 (32.2)	507 (31.9)		382 (30.2)	400 (31.6)	
Histopathological subtypes			0.669			0.957
Metaplastic	1,765 (81.7)	1,321 (83.2)		1,071 (84.7)	1,065 (84.3)	
Adenosquamous	173 (8.0)	117 (7.4)		87 (6.9)	87 (6.9)	
Carcinosarcoma	123 (5.7)	91 (5.7)		63 (5.0)	63 (5.0)	
Adenospindle/cartilaginous/osseus	69 (3.2)	41 (2.6)		31 (2.5)	33 (2.6)	
Epithelial/myoepithelial	30 (1.4)	18 (1.1)		12 (0.9)	16 (1.3)	
Tumor grade			0.558			0.775
Well differentiated	86 (4.0)	67 (4.2)		46 (3.6)	48 (3.8)	
Moderately differentiated	245 (11.3)	188 (11.8)		137 (10.8)	148 (11.7)	
Poorly differentiated	1427 (66.1)	1,068 (67.3)		860 (68.0)	868 (68.7)	
Undifferentiated	100 (4.6)	73 (4.6)		52 (4.1)	52 (4.1)	
Unknown	302 (14.0)	192 (12.1)		169 (13.4)	148 (11.7)	
Tumor size			0.142			0.061
≤10 mm	106 (4.9)	105 (6.6)		52 (4.1)	91 (7.2)	
≤20 mm	373 (17.3)	314 (19.8)		232 (18.4)	275 (21.8)	
≤30 mm	794 (36.8)	511 (32.2)		464 (36.7)	415 (32.8)	
≤40 mm	311 (14.4)	197 (12.4)		200 (15.8)	171 (13.5)	
≤50 mm	189 (8.8)	132 (8.3)		203 (16.1)	218 (17.2)	
>50 mm	387 (17.9)	329 (20.7)				
Lymph node			<0.001			0.419
0	1,463 (67.7)	1,150 (72.4)		1,028 (81.3)	1,013 (80.1)	
1–3	276 (12.8)	223 (14.0)		169 (13.4)	165 (13.1)	
4–9	76 (3.5)	75 (4.7)		45 (3.6)	54 (4.3)	
≥10	345 (16.0)	140 (8.8)		22 (1.7)	32 (2.5)	
Risk stratification			<0.001			<0.001
Low risk	482 (22.3)	402 (25.3)		284 (22.5)	352 (27.8)	
Intermediate risk	1,208 (55.9)	841 (53.0)		727 (57.5)	666 (52.7)	
High risk	257 (11.9)	256 (16.1)		143 (11.3)	175 (13.8)	
Others	213 (9.9)	89 (302)		110 (8.7)	71 (5.6)	
ER status			<0.001			0.253
Negative	1,629 (75.4)	1,230 (77.5)		1,021 (80.8)	1,003 (79.4)	
Positive	355 (16.4)	291 (18.3)		184 (14.6)	211 (16.7)	
Unknown	176 (8.1)	67 (4.2)		59 (4.7)	50 (4.0)	
PR status			<0.001			0.166
Negative	1,743 (80.7)	1,310 (82.5)		1,074 (85.0)	1,052 (83.2)	
Positive	235 (10.9)	205 (12.9)		128 (10.1)	157 (12.4)	
Unknown	182 (8.4)	73 (4.6)		62 (4.9)	55 (4.4)	
Treatment with chemotherapy			<0.001			0.966
Yes	1,078 (49.9)	1,164 (73.3)		874 (69.1)	873 (69.1)	
No	1,082 (50.1)	424 (26.7)		390 (30.9)	391 (30.9)	

MBC, metaplastic breast cancer; ER, estrogen receptor; PR, progesterone receptor; PSM, propensity score matching; PMRT, post-mastectomy radiotherapy.

According to the pathological stage of lymph nodes, we divided lymph node state into four groups by the number of lymph node metastasis.

78.5% of the patients included for this analysis were aged >50 years, with a median age of 63 years (range, 20–89 years). Most of the patients were White (n = 2,850, 76.0%) and had metaplastic NOS (n = 3,086, 82.3%) and poor differentiation (n = 2,495, 66.6%). 67.9% (n = 2545) had access to health insurance. 97.0% (n = 3634) were registered by hospital inpatient/outpatient or clinics. 76.3% (n = 2,859) were ER-negative patients, and 81.5% PR negative (n = 3,053). 2,409 (64.3%) and 799 (21.3%) had tumor size less than 5 cm and greater than 5 cm, respectively. 2,613 (69.7%) exhibited numerous non-metastasized axillary lymph nodes, 42.4% (n = 1,588) received PMRT, and 59.8% (n = 2,242) underwent chemotherapy. Old patients and those with larger tumor sizes, and numerous lymph nodes were more likely to receive PMRT.

### Survival Analyses Before PSM

The median follow-up time for the entire cohort was 42 months, 39 months for the non-PMRT group, and 49 months for the PMRT group. There were 1,300 and 799 patient deaths in the whole cohort and breast cancer related-deaths, respectively.

The univariate analysis performed between groups revealed difference in age [(≤ 50), p < 0.001], race (p = 0.037), lymph node status (p < 0.001), ER status (p < 0.001), and PR status (p < 0.001), but not in tumor sizes; tumor sizes were similar in both groups. The 5- and 10-year OS rates of all the patients were 66.2% and 53.6%, respectively, and the corresponding 5- and 10-year BCSS rates were 75.3% and 71.5%.

**Figure 2:**
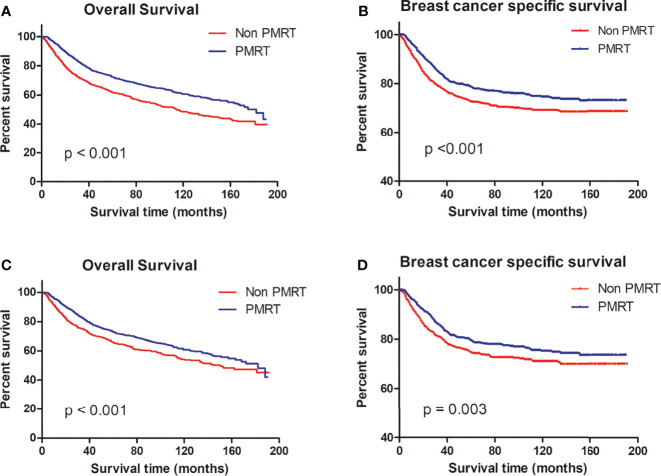
OS and BCSS of MBC patients displayed as Kaplan–Meier curve stratified according to PMRT. **(A)** OS curve of the non-PMRT group versus PMRT group before PSM; **(B)** BCSS curves of the non-PMRT group versus PMRT group before PSM; **(C)** OS curve of the non-PMRT group versus PMRT group after PSM; **(D)** BCSS curves of the non-PMRT group versus PMRT group after PSM. MBC, metaplastic breast cancer; OS, overall survival; BCSS, breast cancer-special survival; PMRT, post mastectomy radiotherapy; PSM, propensity score matching.

Per the OS and BCSS on the Kaplan–Meier curve, patients who received PMRT fared better than non-PMRT-receiving patients (**Figures 2A, B**).

### Survival Analysis After PSM

After using age, tumor grade, tumor size, lymph node state, ER status, PR status, and chemotherapy record as covariates, as shown in **Table 2**, no parameters differed in two groups by univariate analysis.

The multivariate analyses of independent prognostic factors for the OS and BCSS showed that PMRT was an independent prognostic factor; the OS (HR 1.44; 95% CI, 1.24–1.66; p < 0.01) and BCSS (HR 1.42; 95% CI, 1.18–1.70; p < 0.01) of non-PMRT-receiving patients were worse than those of their PMRT-receiving counterparts (**Table 3**). Other parameters, including tumor size, the number of axillary node-positive, and tumor grade, were also independent indicators of the OS and BCSS. We did not find age, race/ethnicity, hormone receptor status, insurance status, and chemotherapy records to be associated with OS or BCSS. For propensity score-matched patients, the 5- and 10-year OS rates were 69.3% and 57.3%, respectively, and the corresponding 5- and 10-year BCSS rates were 77.1% and 73.2%, respectively.

**Table 3:** Multivariate analyses of OS and BCSS for the MBC after PSM.

	OS HR	95% CI/p value	BCSS HR	95% CI/p value
Age, years				
20–30	1.00		1.00	
31–40	1.277	0.49–3.32; p = 0.62	1.22	0.47–3.20; p = 0.68
41–50	1.752	0.71–4.34; p = 0.23	1.42	0.57–3.53; p = 0.45
51–60	1.780	0.72–4.38; p = 0.21	1.46	0.59–3.60; p = 0.42
61–70	2.149	0.87–5.28; p = 0.10	1.55	0.63–3.84; p = 0.34
71–80	3.360	1.36–8.28; p < 0.01	1.83	0.74–4.58; p = 0.19
81–90	5.215	2.09–13.02; p < 0.01	2.43	0.94–6.25; p = 0.07
Race/ethnicity				
American Indian/Alaska Native	1.00		1.00	
Asian or Pacific Islander	0.59	0.24–1.44; p = 0.25	0.42	0.15–1.15; p = 0.09
Blank	0.72	0.31–1.68; p = 0.45	0.59	0.23–1.49; p = 0.26
White	0.62	0.27–1.43; p = 0.26	0.52	0.21–1.30; p = 0.16
Unknown	0.36	0.04–3.04; p = 0.35	0.55	0.06–4.85; p = 0.55
Insurance status				
Insured	1.00		1.00	
Medicaid	1.05	0.81–1.37; p = 0.67	0.93	0.68–1.27; p = 0.65
Uninsured/unknown	1.13	0.96–1.33; p = 0.15	1.05	0.85–1.30; p = 0.66
Tumor grade				
Undifferentiated	1.00		1.00	
Poorly differentiated	0.78	0.57–1.06; p = 0.11	0.71	0.49–1.01; p = 0.06
Moderately differentiated	0.67	0.46–0.98; p = 0.04	0.43	0.26–0.71; p < 0.01
Well differentiated	0.57	0.30–1.08; p = 0.08	0.49	0.20–1.19; p = 0.11
Unknown	0.68	0.48–0.96; p = 0.03	0.53	0.34–0.81; p < 0.01
Tumor size				
≤10 mm	1.00		1.00	
≤20 mm	1.70	0.96–2.30; p = 0.07	1.28	0.57–2.90; p = 0.56
≤30 mm	2.53	1.47–4.36; p < 0.01	2.93	1.36–6.28; p < 0.01
≤40 mm	3.07	1.74–5.41; p < 0.01	3.46	1.58–7.60; p < 0.01
≤50 mm	3.35	1.87–6.00; p < 0.01	4.25	1.91–9.48; p < 0.01
>50 mm	7.93	4.56–13.77; p < 0.01	9.30	4.31–20.07; p < 0.01
Lymph node state				
0	1.00		1.00	
1–3	1.56	1.28–1.90; p < 0.01	1.85	1.47–2.33; p < 0.01
4–9	2.36	1.76–3.16; p < 0.01	2.72	1.96–3.77; p < 0.01
≥10	3.41	2.34–4.97; p < 0.01	4.00	2.65–6.03; p < 0.01
Risk stratification				
Low risk	1.00		1.00	
Intermediate risk	1.979	1.59–2.46; p < 0.01	2.564	1.85–3.55; p < 0.01
High risk	4.652	3.58–6.05; p < 0.01	6.384	4.42–9.22; p < 0.01
Others	4.554	3.43–6.01; p < 0.01	6.683	4.54–9.85; p < 0.01
ER status				
Negative	1.00		1.00	
Positive	0.83	0.64–1.06; p = 0.13	0.85	0.63–1.15; p = 0.29
PR status				
Negative	1.00		1.00	
Positive	0.92	0.70–1.21; p = 0.53	1.09	0.79–1.50; p = 0.61
Treatment with chemotherapy				
Yes	1.00		1.00	
No	1.37	1.16–1.63; p < 0.01	1.13	0.90–1.43; p = 0.28
Post-mastectomy radiotherapy				
Yes	1.00		1.00	
No	1.44	1.24–1.66; p < 0.01	1.42	1.18–1.70; p < 0.01

MBC, metaplastic breast cancer; ER, estrogen receptor; PR, progesterone receptor; PSM, propensity score-matched; PMRT, post-mastectomy radiotherapy; OS, overall survival; BCSS, breast cancer specific survival.

According to the pathological stage of lymph nodes, we divided lymph node state into four groups by the number of lymph node metastasis.

Per the OS and BCSS of matched patient pairs on the Kaplan–Meier curve, patients who received PMRT fared better than non-PMRT-receiving patients (**Figures 2C, D**).

### Subgroup Analysis According to the Risk Stratification

**Figure 3:**
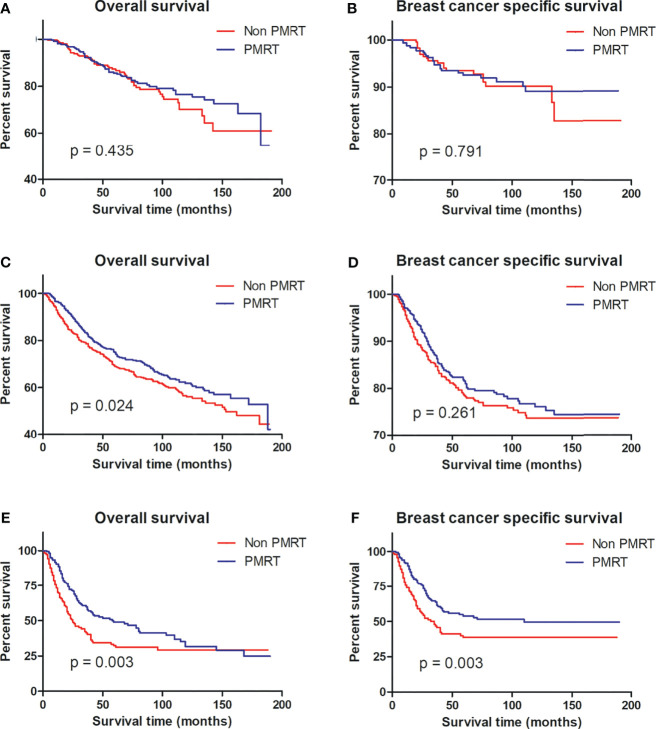
OS and BCSS curves for all patients with MBC with and without PMRT in the low-risk **(A, B)**, intermediate-risk **(C, D)**, and high-risk **(E, F)** groups after PSM. MBC, metaplastic breast cancer; OS, overall survival; BCSS, breast cancer-special survival; PMRT, post mastectomy radiotherapy; PSM, propensity score matching.

To identify that MBC patients benefited from PMRT, subgroup analyses after propensity score matching were performed for low-risk, intermediate-risk, and high-risk MBC. Per Kaplan–Meier analysis, PMRT improved the BCSS of high-risk MBC patients compared to their non-PMRT-receiving counterparts (p = 0.003), while PMRT could not benefit for patients with low- (p = 0.791) and intermediate-risk (p = 0.261) disease. Notably, patients with intermediate- (p = 0.024) and high-risk (p = 0.003) groups did not benefit from PMRT (**Figure 3**).

## Discussion

In our study, we explored the effect of PMRT in MBC and annual percentage change using joinpoint analysis. After propensity score matching and utilizing the reported variables as covariates considered vitally by scholars, we showed that PMRT provided better BCSS in the high-risk groups and better OS in the intermediate- and high-risk groups.

Some researchers have shown that MBC is a rare histologic subtype of breast cancer, as defined by the World Health Organization (WHO) in 2001, and represents approximately 2%–5% of breast cancers diagnosed annually (28–30), while after the year 2001, our results showed that the incidence rates increased with the increase accelerating by 1.99% per year. Those indicated that the current MBC incidence rate could be higher than 5%.

Our propensity score matching revealed that chemotherapy did not improve the OS and BCSS of the MBC patients, consistent with findings from other studies (3, 23, 31–33). Patients with axillary lymph node-negative accounted for 69.7% (n = 2613). Consistent with previous results, MBC patients displayed with axillary lymph node-negative, which was in accord with its sarcomatoid type and its tendency to metastasize through the hematogen rather than the lymph (34, 35). Hormone receptor and HER-2 receptor expressions were lower in MBC cells than in IDC cells, but Ki-67 and p53 levels were higher in MBC cells than in IDC cells (36, 37). DNA repair pathways—TOP2A, PTEN, and BRCA1 pathway—were downregulated by genomic profiling analysis (38, 39), illustrating that MBC could metastasize to the lymph node with low incidence, resist to conventional chemotherapy regimens, and be sensitive to the radiotherapy.

The National Comprehensive Cancer Network breast cancer guidelines recommended that patients with the T1-2N1 stage receive PMRT and those with the N2 stage get PMRT management (40). Additionally, the 5-year survival rates for MBC patients ranged from 49% to 83%, suggesting that the role of PMRT in MBC patients is unclear. In the analysis by Wang et al. on MBC patients’ data from the SEER database between 2000 and 2014 (21), PMRT improved the survival of intermediate- and high-risk MBC patients. Intermediate risk was defined as stage T1-2N1M0 and T3N0M0 (25) and high risk as stage T1-4N2-3M0 and T4N0-1M0 (26). No differences were observed in demographics between the PMRT group and the non-PMRT group without PSM. However, tumor size was an important factor leading to the poor prognosis of MBC. Considering the heterogeneity of the research population, we performed a propensity score-matching cohort to ensure that the better survival observed in MBC patients stemmed from receiving radiotherapy and not from differences in demographics and clinical pathological characteristics. Our further exploration on the effect of radiotherapy in patients with MBC among different subgroups showed that the OS, but not the BCSS, of patients at stage T1-2N1 improved after receiving PMRT. Our conclusion differs from those of previous studies.

In the analysis by Li et al. on clinical-pathologic information, 2,267 MBC patients were registered between 1998 and 2015 in the SEER database (14). In addition, they conducted a subgroup analysis to assess the benefit of PMRT on some parameters, such as age, T stage, and N stage after PSM, and showed that the PMRT group had better survival than the non-PMRT group, and older patients or larger tumors benefited from PMRT. However, MBC was not defined as a unique histologic subtype by WHO until 2000 (1). According to joinpoint regression, the incidence of MBC decreased significantly before 2003, which might have indicated that the diagnosis of MBC was vague for pathologists before 2003, leading to the phenomenon where the MBC incidence was decreasing. After dividing the entire cohort into 2 eras with a cutoff value of 2,000, Warren H. Tseng (15) concluded that the diagnoses of MBC and combined epithelial–mesenchymal histology are similar entities before and after 2000.

In this study, we included all demographics and clinical-pathological characters after 2000 and used PSM to ensure that the better survival observed in MBC patients was a result of radiotherapy. We also stratified the case–control matching patients into low-risk, intermediate-risk, and high-risk groups to find the population more appropriate for PMRT.

Despite all the precautions we took, our research still had a few limitations. First, our data may be incomplete which would influence our results. Second, information on chemotherapy and radiotherapy was scant. Finally, histopathology information from the SEER database was not reviewed.

## Conclusion

Based on our findings, the number of MBC patients in the annual percentage change has been increasing significantly since approximately 2000. However, our analyses show that PMRT could provide better BCSS in high-risk patients and better OS in the intermediate- and high-risk patients.

## Data Availability Statement

The raw data supporting the conclusions of this article will be made available by the authors, without undue reservation.

## Ethics Statement

Ethical review and approval were not required for the study on human participants in accordance with the local legislation and institutional requirements. Written informed consent for participation was not required for this study in accordance with the national legislation and the institutional requirements.

## Author Contributions

JH and JT are lead authors who participated in data collection, manuscript drafting, table/figure creation, and manuscript revision. FD and XZ are senior authors who aided in drafting the manuscript and manuscript revision. TH and JM are the corresponding authors who initially developed the concept and drafted and revised the manuscript. All authors contributed to the article and approved the submitted version.

## Conflict of Interest

The authors declare that the research was conducted in the absence of any commercial or financial relationships that could be construed as a potential conflict of interest.

## Publisher’s Note

All claims expressed in this article are solely those of the authors and do not necessarily represent those of their affiliated organizations, or those of the publisher, the editors and the reviewers. Any product that may be evaluated in this article, or claim that may be made by its manufacturer, is not guaranteed or endorsed by the publisher.

## Abbreviations

MBC, metaplastic breast cancer; IDC, infiltrating ductal carcinoma; PMRT, post mastectomy radiotherapy; ER, estrogen receptor; PR, progesterone receptor; BCSS, breast cancer-special survival; OS, overall survival; NCCN, the National Comprehensive Cancer Network; SEER, the Surveillance, Epidemiology, and End Results; PSM, propensity score matching; APC, annual percentage change; WHO, the World Health Organization.
